# The Naphthalene Catabolic Genes of *Pseudomonas putida* BS3701: Additional Regulatory Control

**DOI:** 10.3389/fmicb.2020.01217

**Published:** 2020-06-05

**Authors:** Irina Pozdnyakova-Filatova, Kirill Petrikov, Anna Vetrova, Alina Frolova, Rostislav Streletskii, Marina Zakharova

**Affiliations:** ^1^Laboratory of Molecular Microbiology, Pushchino Scientific Center for Biological Research of the Russian Academy of Sciences, Federal Research Center, G.K. Skryabin Institute of Biochemistry and Physiology of Microorganisms, Pushchino, Russia; ^2^Laboratory of Plasmid Biology, Pushchino Scientific Center for Biological Research of the Russian Academy of Sciences, Federal Research Center, G.K. Skryabin Institute of Biochemistry and Physiology of Microorganisms, Pushchino, Russia; ^3^Laboratory of Bacteriophage Biology, Pushchino Scientific Center for Biological Research of the Russian Academy of Sciences, Federal Research Center, G.K. Skryabin Institute of Biochemistry and Physiology of Microorganisms, Pushchino, Russia; ^4^Laboratory of Ecological Soil Science, Lomonosov Moscow State University, Moscow, Russia

**Keywords:** aromatic compound, oxygenase, nitrogen, iron, nucleoid-associated proteins, RT-qPCR

## Abstract

*Pseudomonas* microorganisms are used for bioremediation of soils contaminated with petroleum hydrocarbons. The overall remediation efficiency is largely dependent on the presence of macro- and micronutrients. Widely varying concentrations of available nitrogen and iron (Fe) in soils were shown to affect residual hydrocarbons in the course of biodegradation. The regulatory mechanisms of expression of hydrocarbon catabolic genes in low nitrogen/low iron conditions remain unclear. The catabolism of naphthalene, a two-ring polycyclic aromatic hydrocarbon, has been well studied in pseudomonads in terms of the involvement of specific transcriptional activators, thus making it useful in revealing additional regulatory control of the adaptation of hydrocarbon destructors to a low level of the essential nutrients. The *Pseudomonas putida* strain BS3701 is a component of the “MicroBak” preparation for soil remediation. Previously, this strain was shown to contain genes encoding the key enzymes for naphthalene catabolism: naphthalene 1,2-dioxygenase, salicylate hydroxylase, catechol 2,3-dioxygenase, and catechol 1,2-dioxygenase. Our study aimed to clarify whether the naphthalene catabolic gene expression is dependent on the amount of nitrogen and iron in the growth culture medium, and if so, at exactly which stages the expression is regulated. We cultivated the strain in low nitrogen/low iron conditions with the concurrent evaluation of the activity of the key enzymes and the mRNA level of genes encoding these enzymes. We are the first to report that naphthalene catabolic genes are subject not only to transcriptional but also post-transcriptional regulation.

## Introduction

Microorganisms of the genus *Pseudomonas* are used for bioremediation of soils contaminated with petroleum hydrocarbons. The overall remediation efficiency is largely dependent on the presence of macro- and micronutrients. Widely varying concentrations of available nitrogen and iron (Fe) in soils (Jin et al., 2014; Wang et al., 2018) were shown to affect residual hydrocarbons in the course of biodegradation (Simarro et al., 2011). The regulatory mechanisms of expression of hydrocarbon catabolic genes in low nitrogen/low iron conditions remain unclear. The catabolism of naphthalene, a two-ring polycyclic aromatic hydrocarbon, has been well studied in pseudomonads in terms of the involvement of specific transcriptional activators, thus making it useful in revealing additional regulatory control of the adaptation of hydrocarbon destructors to a low level of the essential nutrients.

The naphthalene transformation to Krebs cycle intermediates was first studied using the *Pseudomonas putida* PpG1 (Yen and Gunsalus, 1982), also known as the *P. putida* strain G7 (pNAH7), where the naphthalene conversion proceeded via salicylate (*nah-*operon, *nahABCDEF*) and catechol further oxidized by the meta-pathway (*sal-*operon, *nahGHIJKLM*). Activation of the *nah-* and *sal*-operons occurred with the assistance of the transcriptional regulator NahR (LysR-type transcriptional regulator, LTTR) in the presence of the salicylate ion (Schell and Poser, 1989). Further studies revealed other variants of the genetic organization of naphthalene catabolic genes (Boronin and Kosheleva, 2010), while genes of the catechol oxidation (by both meta- and ortho- pathways) were described in detail as members of other operons (Ladino-Orjuela et al., 2016). The effect of NahR is frequently believed to apply to both the salicylate transformation genes and genes involved in the naphthalene catabolism [e.g., the “classical” scheme of regulation of naphthalene catabolic genes (Schell and Poser, 1989)], although another study has shown that the mRNA level of naphthalene 1,2-dioxygenase remains unchanged in the presence of salicylate (Boronin and Kosheleva, 2010). The protein CatR is known to be involved in the regulation of genes encoding catechol 1,2-dioxygenase (Tover et al., 2000), with the transcription activation induced by the intermediate *cis,cis*-muconate (Parsek et al., 1992). When considered in the context of naphthalene catabolism, catechol 2,3-dioxygenase is frequently described as non-inducible. However, it is known that the protein XylS that regulates the activity of the toluene/xylene catabolic gene cluster *xylDLEGF* (the *xylE* gene encodes catechol 2,3-dioxygenase) acts as an activator in the presence of toluene and meta-benzyl alcohol (Inouye et al., 1987). The level of *xylS* mRNA depends on XylR which in turn is associated with sigma 54-dependent promoters (Kessler et al., 1994). The nucleoid-associated proteins (NAPs) classified as pleiotropic regulators are also reported to be involved. The integration host factor (IHF) binding site was detected near XylR (sigma-54-dependent Fis family transcriptional regulator that controls toluene/xylene catabolism); the IHF structure appears to be important for transcription activation (Ramos et al., 1997).

The *P. putida* strain BS3701 is a component of the “MicroBak” preparation for soil remediation (Filonov et al., 2006). Previously, this strain was shown to contain genes encoding the key enzymes for naphthalene catabolism: naphthalene 1,2-dioxygenase, salicylate hydroxylase, catechol 2,3-dioxygenase, and catechol 1,2-dioxygenase (Izmalkova et al., 2006). Our study aimed to clarify whether the naphthalene catabolic gene expression is dependent on the amount of nitrogen and iron in the growth culture medium, and if so, at exactly which stages the expression is regulated. We cultivated the strain in low nitrogen/low iron conditions with the concurrent evaluation of the activity of the key enzymes and the mRNA level of genes encoding these enzymes. The RT-qPCR technique was validated for the chosen growth conditions. Using BestKeeper, the *oprI* gene was chosen as the reference gene. We are the first to report that naphthalene catabolic genes are subject not only to transcriptional but also post-transcriptional regulation.

## Materials and Methods

### Growth Media and Conditions

The *P*. *putida* strain BS3701 used in this study was grown at 26°C on modified Evans mineral medium supplemented either with glucose (10 g/l) or sodium salicylate (1 g/l) as the sole source of carbon and energy. The modified Evans mineral medium contained (per liter) 50 mM K_2_HPO_4_, 5 mM NH_4_Cl, 0.1 mM Na_2_SO_4_, 0.0625 mM MgCl_2_, 0.018 mM FeCl_3_, 0.01 mM MnCl_2_, 0.005 mM ZnO, 0.002 mM CoCl_2_, 0.001 mM CaCl_2_, 0.97 μM CuCl_2_, 0.97 μM H_3_BO_3_, and 0.005 μM (NH_4_)_6_Mo_7_O_24_ (Petrikov et al., 2013). The medium pH was adjusted to 7.5 using concentrated HCl. The low nitrogen growth medium contained 1 mM versus 5 mM of NH_4_Cl and was supplemented with sodium salicylate (1 g/l) as the sole source of carbon. The low iron growth medium contained 100 μM 2,2′-bipyridyl (Sigma-Aldrich, United States) and was supplemented with sodium salicylate (1 g/l) as the sole source of carbon. BS3701 grown on modified Evans with glucose (10 g/l) for 1 day was further used for inoculation (1 ml per 100 ml of medium). The harvested cells were centrifuged at 10,000 rpm for 10 min at 4°C and re-suspended in appropriate modified Evans at the final concentration of 3 × 10^8^ CFU/ml. The 750-ml flasks used in the experiments contained the appropriate mineral medium and substrate (100 ml each). The growth period lasted until the end of the exponential growth phase. The time of cultivation on all used media was 31 h, the end of exponential growth phase was 21 h.

### Determination of the Oxidative Activity of Microorganisms in Relation to Sodium Salicylate

The oxidative activity (OA) of microorganisms was determined from sodium salicylate degradation vs. control (microorganism-free medium):

$$\text{OA}=\frac{{\text{X}}_{\text{k}}-{\text{X}}_{\text{i}}}{{\text{X}}_{\text{k}}\times 100\%}$$

where X_*k*_ is sodium salicylate concentration (mg/l) in the microorganism-free medium and X_*i*_ is that in the inoculated flask (mg/l). Residual concentration of sodium salicylate (20 μl sample) was analyzed by liquid chromatography using a silica gel-packed Agilent (1260 infinity) column (length 150 mm, diameter 4 mm, grain size 5 μm) grafted with hexadecyl groups. The used wavelength was 310 nm. The mobile phase was (A) water (0.1% CH_3_COOH); (B) acetonitrile (0.1% CH_3_COOH). The gradient mode was as follows: up to 3 min – 20% of eluent B, by 12 min – 50% of eluent B, by 14 min – 100% of eluent B. The eluent flow rate was 1 ml/min. System stabilization took 5 min; the analysis time was 19 min (Liu and Smith, 1996). The column thermostat temperature was 40°C. For sample preparation, 2 ml of the culture was taken from the flask and centrifuged for 10 min at 10,000 rpm. For the measurements, 0.5 ml samples of supernatant were used. If necessary, the supernatant was diluted with distilled water and analyzed as described above. Standards were prepared in distilled water by serial dilution.

### Measuring Enzymatic Activity

The biomass was washed twice with chilled 0.05 M phosphate buffer (pH 7.0) and re-suspended in 0.02 M phosphate buffer (pH 7.5). Cell suspension (5 ml sample) was subjected to ultrasonic disintegration (using an MSE150 disintegrator) for 1.5 min (3 × 30 s) at 4°C. Cell debris was removed by centrifugation (a Rotanta 460R centrifuge, Hettich Zentrifugen, Germany) for 20 min at 32,000 × *g* and 4°C. The supernatant was used as a cell-free extract in measuring enzymatic activity.

Activities of the enzymes were determined as previously described for naphthalene 1,2-dioxygenase in Dua and Meera (1981), for salicylate hydroxylase in Yamamoto et al. (1965), for catechol 1,2-dioxygenase in Hegeman (1966), and catechol 2,3-dioxygenase in Feist and Hegeman (1969). The protein concentration was determined according to Bradford (1976).

### RT-qPCR

Specific primers were constructed using the Primer-BLAST tool^1^ (Table 1). A kit containing SYBR Green (cat. no. R-402, Sintol, Russia) was used for qPCR. For amplification, the following temperature program was used: (1) 95°C for 3 min, (2) 95°C for 20 s, (3) 60°C for 20 s, (4) 72°C for 5 s; 40 cycles included steps (2–4). For each pair of primers, amplification efficiency was determined from the slope of the log-linear portion of the calibration curve. The reaction specificity was confirmed by analysis of the melting curve and by agarose gel electrophoresis Total RNA was isolated using the TRI reagent according to the manufacturer’s instructions (Sigma-Aldrich, United States). A RevertAid RT Reverse Transcription Kit (Thermo, United States) was used according to the manufacturer’s protocol for the reverse transcription reaction including 100 ng of total RNA and specific primers (20 pmol of each reverse primer). To validate this method, a reference gene was selected among *16S rRNA*, *rpoD, rpoB, rpoS, gyrB, tuf, ppiD, oprI, rpsL, dnaK, fliS*, and *proC*. According to BestKeeper (Pfaffl et al., 2004), the most stable was the *oprI* gene (SD 0.69, CV 9.8%). The relative amount of transcripts was quantified using the delta-delta Cp method, with amplification efficiency taken into account:

**TABLE 1:** Primers for quantitative reverse transcription PCR.

Gene	Sequence, 5′-3′	Amplicon length, bp	PCR efficiency	References
*16S rRNA*	16S1	207	2.26	Li S. et al., 2011
	CGGGAATCTGACACAGGTGCT			
	16S2			
	GATCCGGACTACGATCGTTTTGT			
*rpoD*	rpoD(BS3701)RT788f	161	2.10	This study
	AGCTGGTACCGAAGCAGTTC			
	rpoD(BS3701)RT948r			
	CTGGTCGGTTTCGTTGCTTG			
*rpoB*	rpoB(BS3701)RT2273f	220	2.03	This study
	TGACCAAGTACACCCGTTCG			
	rpoB(BS3701)RT2492r			
	TCTTCCTGAACCACACGCTC			
*rpoS*	rpoS(BS3701)RT861f	107	1.95	This study
	AGGGCATGAAAGCAGTACCC			
	rpoS(BS3701)RT967r			
	GGATCTCACGCAAACGCTTC			
*gyrB*	gyrB(BS3701)RT1585f	214	2.05	This study
	ATCTATATTGCCCAGCCGCC			
	gyrB(BS3701)RT1798r			
	ACAGGCGCTTGAGAGTCTTC			
*tuf*	tuf(BS3701)RT512f	221	1.98	This study
	TCATCGGTTCGGCTCGTATG			
	tuf(BS3701)RT732r			
	ATCCTGAACGCGGACGATAC			
*ppiD*	ppiD(BS3701)RT890f	220	1.95	This study
	GTGAGGACTTTGCCGCTTTG			
	ppiD(BS3701)RT1109r			
	AAGCTCGGTACTTCTGGTGC			
*oprI*	oprI(BS3701)RT97f	71	1.92	This study
	GAATCAGCGGAGAACAACGC			
	oprI(BS3701)RT167r			
	ACCAGATCGATGCGGTAACA			
*rpsL*	rpsL(BS3701)RT148f	171	2.07	This study
	CGTAAAGTATGCCGTGTGCG			
	rpsL(BS3701)RT318f GCCCGAAGTATCCAGAGAGC			
*dnaK*	dnaK(BS3701)RT256f	212	1.91	This study
	ATCAAGCTGGTGCCGTACAA			
	dnaK(BS3701)RT467r			
	TCTTTGGTAGCCTGACGCTG			
*fliS*	fliS(BS3701)RT288f	80	1.95	This study
	CGAAGCCAATCTCAAAGGCG			
	fliS(BS3701)RT367r			
	CGTCCCAGCCTTCCTTTACA			
*proC*	proC(BS3701)RT267f	148	1.81	This study
	TGGCCAGCTGATCGTTTCAA			
	proC(BS3701)RT414r			
	CACTTCAGCGGTAGCGTACA			
*nahAa*	nahAa(BS3701)RT202f	145	2.21	This study
	CAGTATGTGCTCGCCTGTCA			
	nahAa(BS3701)RT346r			
	AGCGACGGATATCGTGAGTG			
*nahU*	nahU(BS3701)RT279f	112	2.40	This study
	CAAATACCTCGGTTGCAGCG			
	nahU(BS3701)RT390r			
	CCCGAACTGGGCAATACCTT			
*nahH*	nahH(BS3701)RT694f	129	2.03	This study
	ATCAGCATGACCGACACCTC			
	nahH(BS3701)RT822r			
	ATAGTTGTAGTTCCCGCCGC			
*catA*(0)	catA(0)(BS3701)RT344f	174	1.88	This study
	CGATTGCCCAAGGTGAAGTG			
	catA(0)(BS3701)RT517r			
	ACTGGCTTGGGTCGAAGAAC			
*catA*(2)	catA(2)(BS3701)RT548f	85	1.85	This study
	CCGATGATCAAGGGCGCTAT			
	catA(2)(BS3701)RT632r			
	AGG CAT TCC TGA GTA GGG C			
*catA*(8)	catA(8)(BS3701)RT387f	155	2.28	This study
	AGGCGTGGTGATGTTCCTTC			
	catA(8)(BS3701)RT541r			
	TAATGATACGCCGACGCAGG			
*ihfA*	ihfA(BS3701)RT84f	159	2.20	This study
	GCACGCACTTGAAGAGAACG			
	ihfA(BS3701)RT242r			
	TTCAACTTCTGCCCTGGACG			
*ihfB*	ihfB(BS3701)RT138f	135	1.90	This study
	CGGTTTTGGCAGCTTCTCGC			
	ihfB(BS3701)RT272r			
	TTGACCCGATCACGCAGCTC			
*fis*	fis(BS3701)RT122f	85	2.15	This study
	TGACGGACGTGTACAACCTG			
	fis(BS3701)RT206r			
	GTCTGGTTGCCCTTCACGTA			
*lrp*	lrp(BS3701)RT253f	158	2.32	This study
	TCAGGCGATACCTTCGAGGA			
	lrp(BS3701)RT410r			
	AGTTTCAGCAGGATGTCCCC			
*iciA*	iciA(BS3701)RT36f	79	1.93	This study
	AGTGATCGAACAAGGCGGTT			
	iciA(BS3701)RT114r			
	CTTGATGCGCTGGGAAATGG			
*mvaT*10	mvaT10(BS3701)RT280f	83	2.00	This study
	GTGATCGAAACCAAAGGCGG			
	mvaT10(BS3701)RT362r			
	GCCCAGCTTTCAACCACATC			
*mvaT*22	mvaT22(BS3701)RT51f	209	1.93	this study
	ACTTCAACAGCTGGATGCGA			
	mvaT22(BS3701)RT259r			
	TCTTGTAGCGCTTGACCTCG			
*mvaT*34	mvaT34(BS3701)RT275f	86	1.97	This study
	TCATCGAGACCAAAGGTGGC			
	mvaT34(BS3701)RT360r			
	GGAAACCCAAGACTCGACCA			
*mvaT*39	mvaT39(BS3701)RT231f	126	1.93	This study
	GCGGGTGGTCAAGGTCTATC			
	mvaT39(BS3701)RT356r			
	CGTACCCAGCTTTCTACCGT			
*mvaT*41	mvaT41(BS3701)RT130f	185	1.93	This study
	CTGGCCAAGTACGGCTACAG			
	mvaT41(BS3701)RT314r			
	TCCTTCAATCGCGAGTGGTT			

$$\text{Value=}\frac{E_{gene of interest}{\;}^{\left({\text{C}}_{\text{p}\;gene of interest.control}-{\text{C}}_{p gene of interest.experimental}\right)}}{E_{reference gene}{\;}^{\left({\text{C}}_{p reference gene.control}-{\text{C}}_{p reference gene.experimental}\right)}}$$

where «E» is the amplification efficiency, «Cp» is the crossing point. We used unpaired Student’s *t*−test for data comparison, *p*-value < 0.05. All results are derived from three independent replicates and presented as a mean score ± confidence interval.

### References to NCBI Databases

The nucleotide sequences of the naphthalene catabolic genes have been deposited in GenBank under accession nos. MN442422.1, MN413629.1, MN442423.1, MN442424.1, MN442425.1, and MN442426.1.

## Results

### Naphthalene-to-Salicylate Transformation

**FIGURE 1:**
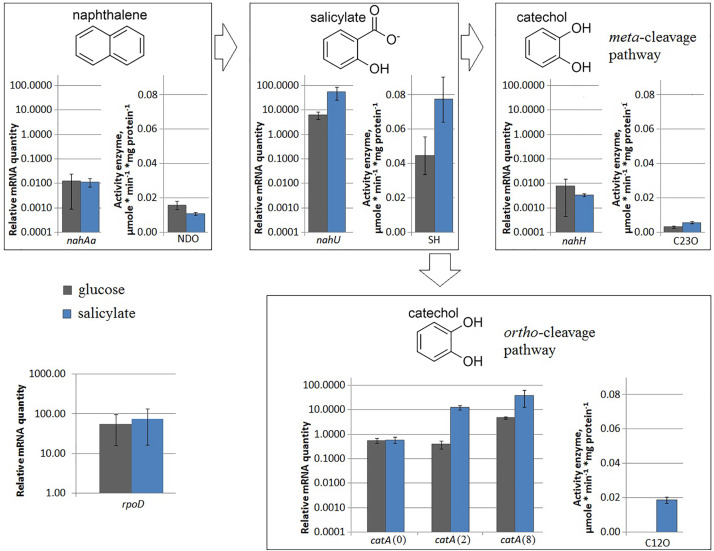
The relative level of mRNA and enzymatic activity in *Pseudomonas putida* BS3701 grown on Evans supplemented either with glucose (dark-gray bars) or salicylate (blue bars). *rpoD*, sigma-70-dependent RNAP subunit; *nahAa*, reductase component of naphthalene dioxygenase; *nahU*, salicylate hydroxylase; *nahH*, catechol 2,3-dioxygenase; *catA*(0), *catA*(2) and *catA*(8), catechol 1,2-dioxygenase; NDO, activity of naphthalene 1,2-dioxygenase; SH, activity of salicylate hydroxylase; C23O, activity of catechol 2,3-dioxygenase; C12O, activity of catechol 1,2-dioxygenase. The experiments were performed in three biological replicates, the data are presented as a mean score ± confidence interval (*p*-value < 0.05).

In the *P. putida* strain BS3701, the transformation of naphthalene to salicylate is assisted by the plasmid-localized gene cluster *nahAaAbAcAdBFCQED*. BS3701 cultivation in salicylate-supplemented (vs. glucose) Evans medium (Figure 1) did not result in an altered *nahAa* mRNA level [in contrast to the case of *P. putida* G7 (pNAH7)] but showed an ∼1.5-fold decrease in naphthalene 1,2-dioxygenase activity (NDO) (from 0.016 ± 0.003 to 0.0107 ± 0.0008 μmol/min^∗^mg of protein).

**FIGURE 2:**
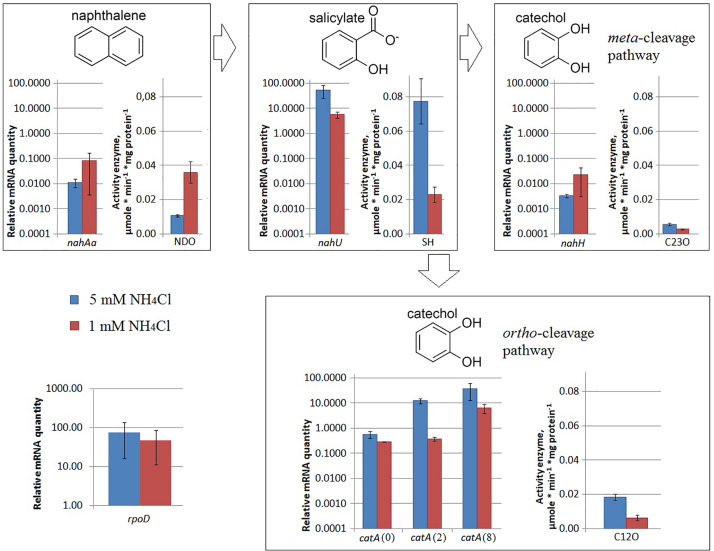
The relative level of mRNA and enzymatic activity in *Pseudomonas putida* BS3701 grown on Evans with 5 mM NH_4_Cl (blue bars) or 1 mM NH_4_Cl (red bars). Each medium was supplemented with salicylate. *rpoD*, sigma-70-dependent RNAP subunit; *nahAa*, reductase component of naphthalene dioxygenase; *nahU*, salicylate hydroxylase; *nahH*, catechol 2,3-dioxygenase; *catA*(0), *catA*(2) and *catA*(8), catechol 1,2-dioxygenase; NDO, activity of naphthalene 1,2-dioxygenase; SH, activity of salicylate hydroxylase; C23O, activity of catechol 2,3-dioxygenase; C12O, activity of catechol 1,2-dioxygenase. The experiments were performed in three biological replicates, the data are presented as a mean score ± confidence interval (*p*-value < 0.05).

Under nitrogen limitation, the *nahAa* mRNA level remained unchanged, while the NDO enzyme activity changed (Figure 2). Evans-cultivated BS3701 in low nitrogen conditions (1 mM NH_4_Cl instead of 5 mM NH_4_Cl) retained the same *nahAa* mRNA level, but the NDO activity increased by ∼3.4-fold (from 0.0107 ± 0.0008 to 0.036 ± 0.006 μmol/min^∗^mg of protein). Previously, the literature offered no reports on the stimulation of NDO synthesis by low nitrogen.

**FIGURE 3:**
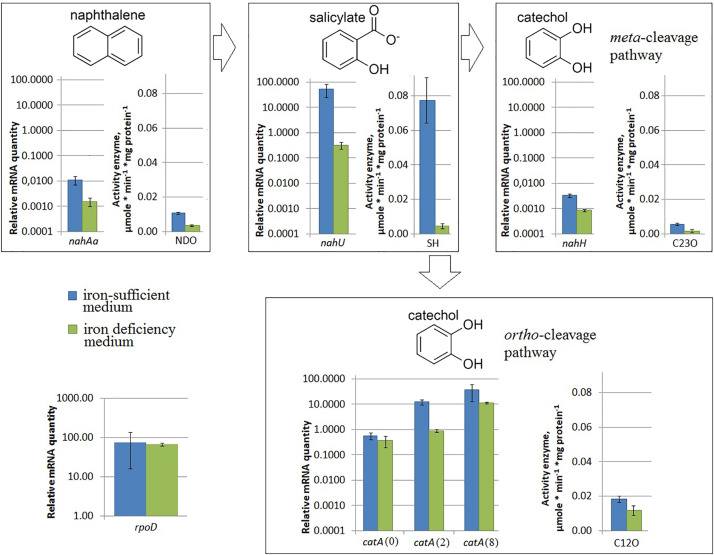
The relative level of mRNA and enzymatic activity in *Pseudomonas putida* BS3701 grown on Evans with a sufficient iron content (blue bars) or low-iron Evans (green bars). Each medium was supplemented with salicylate. *rpoD*, sigma-70-dependent RNAP subunit; *nahAa*, reductase component of naphthalene dioxygenase; *nahU*, salicylate hydroxylase; *nahH*, catechol 2,3-dioxygenase; *catA*(0), *catA*(2), and *catA*(8), catechol 1,2-dioxygenase; NDO, activity of naphthalene 1,2-dioxygenase; SH, activity of salicylate hydroxylase; C23O, activity of catechol 2,3-dioxygenase; C12O, activity of catechol 1,2-dioxygenase. The experiments were performed in three biological replicates, the data are presented as a mean score ± confidence interval (*p*-value < 0.05).

Under iron limitation, both the *nahAa* mRNA level and the NDO enzyme activity changed (Figure 3). Evans-cultivated BS3701 in low iron conditions (with added 100 μM 2,2′-bipyridyl) showed a decrease of the *nahAa* mRNA level by ∼7-fold, and a decrease in the NDO activity by ∼3-fold (from 0.0107 ± 0.0008 to 0.0036 ± 0.0006 μmol/min^∗^mg of protein).

### Salicylate-to-Catechol Transformation

The *P. putida* BS3701 chromosome was found to carry the functional *nahU* gene encoding salicylate hydroxylase (SH). BS3701 cultivation in Evans medium supplemented with salicylate (vs. glucose) (Figure 1) increased the mRNA level by ∼9-fold and the SH activity by ∼1.7-fold (from 0.045 ± 0.011 to 0.077 ± 0.013 μmol/min^∗^mg of protein).

Under nitrogen limitation, both the *nahU* mRNA level and the SH enzyme activity changed (Figure 2). Evans-cultivated BS3701 in low nitrogen conditions showed a decrease in the *nahU* mRNA level by ∼9.6-fold and in the SH activity by ∼3.3-fold (from 0.077 ± 0.013 to 0.023 ± 0.005 μmol/min^∗^mg of protein). The extent of salicylate degradation was also reduced (Table 2). The initial concentration of sodium salicylate in the medium was 1000 mg/L. After BS3701 cultivation in Evans supplemented with 5 mM NH_4_Cl, salicylate was not detected at the end of the exponential growth phase (the residual content was <0.3 mg/l). However, similar to cultivation in 1 mM NH_4_Cl Evans, the residual content of salicylate was 4.3 ± 0.6 mg/L.

**TABLE 2:** The residual salicylate content (for details, see section “Materials and Methods”).

Medium*	Concentration of sodium salicylate at the end of the exponential growth phase, mg/l
Without deficiency	non detected
Low nitrogen	4.3 ± 0.6
Low iron	69 ± 18

*Initial concentration of sodium salicylate was 1000 mg/l.

Under iron limitation, both the *nahU* mRNA level and the SH enzyme activity changed (Figure 3). Evans-cultivated BS3701 in low iron conditions showed an approximately 170-fold *nahU* mRNA decrease with ∼15.4-fold lower activity of SH (from 0.077 ± 0.013 to 0.0050 ± 0.0010 μmol/min^∗^mg of protein). At the end of the exponential growth phase, the residual salicylate content was 69 ± 18 mg/L (Table 2). The literature describes salicylate-derived siderophores, such as *Pseudomonas* pyochelin (Crosa and Walsh, 2002) and promysalin (Li W. et al., 2011). As revealed using RAST and Prokka, BS3701 contains homologs of genes involved in the synthesis and transport of pyochelin (*fptA, pchR, pchB*; unpublished data).

### Meta-Cleavage Pathway of Catechol Degradation

*Pseudomonas putida* BS3701 was found to contain a plasmid-localized gene encoding catechol 2,3-dioxygenase (C23O). During BS3701 cultivation in the presence of salicylate used as a source of carbon (Figure 1), the *nahH* mRNA level remained unchanged, while the C23O activity increased by ∼1.8-fold (from 0.031 ± 0.008 to 0.057 ± 0.007 μmol/min^∗^mg of protein).

Under nitrogen limitation, the *nahH* mRNA level remained unchanged, while the C23O enzyme activity changed (Figure 2). Evans-cultivated BS3701 in low nitrogen conditions showed no change in the *nahH* mRNA level but the C23O decreased by ∼2-fold (from 0.057 ± 0.007 to 0.028 ± 0.003 μmol/min^∗^mg of protein). To date, the literature presents no data on the regulatory mechanisms of expression of genes encoding aromatic ring-hydroxylating dioxygenases in low nitrogen conditions. Under iron limitation, both the *nahH* mRNA level and the C23O enzyme activity changed (Figure 3). Evans-cultivated BS3701 in low iron conditions showed a decrease in the *nahH* mRNA level by ∼3.7-fold and C23O activity by ∼3.3-fold (from 0.057 ± 0.007 to 0.0175 ± 0.0009 μmol/min^∗^mg of protein).

### Ortho-Cleavage Pathway of Catechol Degradation

The *P. putida* BS3701 genome contains three copies of the gene encoding catechol 1,2-dioxygenase (C12O) that are localized to the chromosome and denoted as *catA*(0), *catA*(2), and *catA*(8). Although the *catA*(2) gene is in the immediate vicinity of *nahU* (the distance between the nucleotide A of the *catA*(2) start codon ATG and the nucleotide A of the *nahU* stop-codon TGA is 74 bp), it apparently has its own promoter. In BS3701 grown on Evans supplemented with salicylate (vs. glucose) (Figure 1), the level of *catA*(0) mRNA remained unchanged, while the content of *catA*(2)*-* and *catA*(8) mRNA increased by ∼32-fold (from 0.39 ± 0.15 to 12.46 ± 3.11) and ∼8-fold (from 4.8 ± 0.6 to 38.2 ± 28.4), respectively. C12O cultivated in the presence of glucose showed no activity which was observed only with salicylate used as a source of carbon (0.0186 ± 0.0018 μmol/min^∗^mg of protein).

Under nitrogen limitation, the *catA* mRNA level remained unchanged, while the C12O enzyme activity changed (Figure 2). BS3701 growth on low nitrogen Evans resulted in lower mRNA levels of all three *catA* copies: for *catA*(0) it was an approximately 2-fold decrease (from 0.58 ± 0.19 to 0.29 ± 0.01), for *catA*(2) ∼33-fold (from 12.46 ± 3.11 to 0.38 ± 0.07), and for *catA*(8) ∼6-fold (from 38.2 ± 28.4 to 6.5 ± 2.8). Though the decrease extents were different, the tendency was the same, reflecting the regulation of catechol 1,2-dioxygenase expression as early as at the level of the varying mRNA content. The activity of C12O was ∼3 times lower (from 0.0186 ± 0.0018 to 0.0064 ± 0.0016 μmol/min^∗^mg of protein).

Under iron limitation, the *catA* mRNA level changed for 2 out of 3 copies, and the C23O enzyme activity changed as well (Figure 3). Evans-cultivated BS3701 in low iron conditions retained the same *catA*(0) mRNA level and showed a decrease in *catA*(2)*-* and *catA*(8) mRNAs by ∼13.5- (from 12.46 ± 3.11 to 0.92 ± 0.15) and ∼33-fold (from 38.2 ± 28.4 to 11.5 ± 0.9), respectively. The activity of C12O decreased by ∼1.5-fold (from 0.0186 ± 0.0018 to 0.012 ± 0.003 μmol/min^∗^mg of protein).

### Bioinformatics Analysis of Regulatory Regions in Genes

**FIGURE 4:**
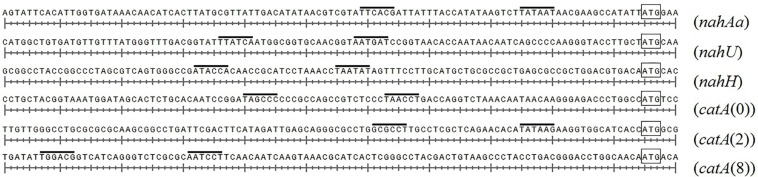
The structural organization of the regulatory region of gene clusters containing *nahAa, nahU, nahH, catA*(0), *catA*(2), and *catA*(8). The putative –10 and –35 promoter boxes are overlined, the start codon (ATG) closest to the promoter is boxed.

Figure 4 presents DNA fragments with putative promoters of naphthalene catabolic genes. Sigma-70-dependent promoters of the *nahAaAbAcAdBFCQED* operon, the *nahU* gene, and *nahH*-containing gene clusters were identified using BPROM, a SoftBerry prediction program. Localization of the putative promoter of a *catA*(0)-containing gene cluster was defined using the literature data. Upstream of the *benA* gene [which probably shares the sole operon with the *catA*(0) gene] resides a sequence that is 98% identical to the regulatory region adjacent to +1 point of the *benABCDKEF* operon in *P. putida* PRS2000 (Cowles et al., 2000). The regions located upstream of the *catA*(2) gene exhibit homology, though extremely low, to the −10 and −35 boxes of the sigma-70-dependent promoter. Since the *catA*(2) mRNA content was observed to increase in cells grown in the presence of salicylate, it cannot be ruled out that the transcription activation is mediated by a specific transcription factor that enables RNA polymerase (RNAP) to recognize such a weak promoter. The promoter of the *catA*(8)-containing *catBCA* operon is identical to that identified for *P. putida catBCA* (Chugani et al., 1997). Using the online analyzer Virtual Footprint, the regulatory regions (from ATG to approximately −60 of the putative promoter) were found to have binding patterns of the nucleoid-associated proteins (NAPs) Lrp, Fis, IHF, H-NS, and IciA. Table 3 presents mean scores ± confidence interval typical of operators of these NAPs, as well as scores for operators revealed in the regulatory region of naphthalene catabolic genes.

**TABLE 3:** Nucleoid-associated proteins with binding patterns located in the studied regulatory regions.

	Mean score ± standard deviation	*nahAa*	*nahU*	*nahH*	*benA* [*catA*(0)]	*catA*(2)	*catB* [*catA*(8)]
Lrp	5.79 ± 0.41	3.55	4.48	4.59	6.77	5.01	4.49
		3.52	4.43	4.41	5.70	4.74	4.17
		3.51	4.39	4.40	5.57		4.15
Fis	3.56 ± 0.31	2.55	3.15	3.31	3.1	2.95	3.37
		2.47	3.11	3.22	3.0	2.91	3.20
			3.01	3.17	2.9	2.84	3.12
IHF	7.67 ± 0.36	–	5.79	–	–	6.24	6.79
						5.86	6.05
H-NS	6.73 ± 0.07	5.27	5.72	–	–	5.27	5.39
			5.58			5.22	
			5.25			5.04	
IciA	5.73 ± 0.09	5.01	4.94	–	5.66	5.16	5.04
		4.64	4.89				
			4.77				

*the *nahAa* region is upstream of the gene encoding reductase component of naphthalene dioxygenase; the *nahU* region is upstream of the gene encoding salicylate hydroxylase; the *nahH* region is upstream of the *nahH* gene; the *benA* [*catA*(0)] region is upstream of the *benA* gene of the cluster containing the catechol 1,2-dioxygenase *catA*(0) gene; the *catA*(2) region is upstream of the catechol 1,2-dioxygenase *catA*(2) gene; the *catB* [*catA*(8)] region is upstream of the *catB* gene of the cluster containing the catechol 1,2-dioxygenase *catA*(8) gene. The tabulated values are mean scores ± confidence interval typical for each regulator according to Virtual Footprint (the column « mean score ± standard deviation»). For each regulatory region, Table presents scores of all detected binding patterns of each NAP.*

The *P. putida* BS3701 genome contains one copy of genes coding for Fis (factor for inversion stimulation), Lrp (leucine responsive protein), and IciA (inhibitor of chromosome initiation), five copies of the gene coding for H-NS (histone-like nucleoid-structuring protein, MvaT for *Pseudomonas*, 50–88% homology of amino acid sequences), as well as one copy of genes coding for alpha- and beta-subunits of IHF (integration host factor) (unpublished data).

### Changes in the mRNA Level of *rpoD* and NAPs

**FIGURE 5:**
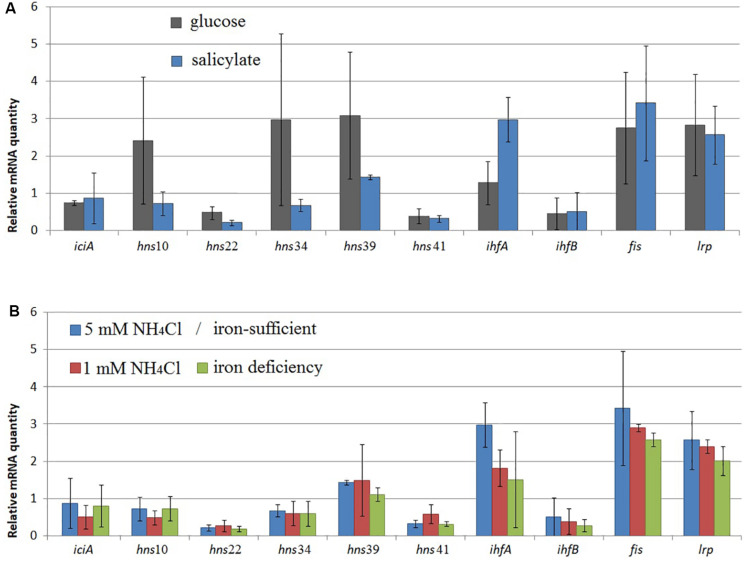
The relative mRNA level of genes encoding NAPs in *Pseudomonas putida* BS3701 grown: **(A)**, on glucose-supplemented Evans (dark-gray bars) or salicylate-supplemented Evans (blue bars); **(B)**, on Evans (Evans, blue bars), low-nitrogen Evans (red bars), and low-iron Evans (green bars). *iciA*, inhibitor of chromosome initiation; *mvaT*, histone-like nucleoid-structuring protein (*mvaT*10, 22, 34, 39, and 41 are homologs); *ihfA* and *ihfB*, alpha- and beta-subunits of integration host factor, respectively; *fis*, factor for inversion stimulation; *lrp*, leucine responsive protein. The experiments were performed in three biological replicates, the data are presented as a mean score ± confidence interval (*p*-value < 0.05).

A change was observed in the mRNA level of *rpoD* encoding RNAP σ70-subunit (Figures 1–3) and in that of *iciA, mvaT, ihfA, ihfB, fis*, and *lrp* (Figure 5). Cultivation of BS3701 in Evans supplemented with salicylate (vs. glucose) (Figure 5A) resulted in a statistically significant increase of mRNA of the gene encoding the IHF alpha subunit (by ∼2.3-fold, from 1.28 ± 0.57 to 3.0 ± 0.6). IHF can stimulate the interaction between RNAP and specific translation regulators (Hoover et al., 1990). The literature presents no information concerning the change in IHF amount in response to salicylate. The IHF involvement in the regulation of naphthalene catabolic gene expression certainly requires further experimental confirmation.

BS3701 grown on Evans in low nitrogen conditions demonstrated (Figure 5B) decreasing mRNA of the gene encoding IHFa (by ∼1.6-fold, from 3.0 ± 0.6 to 1.8 ± 0.5), whereas the literature describes IHF as a co-activator of the transcription of genes involved in nitrogen fixation (Hoover et al., 1990).

Evans-cultivation of BS3701 in low iron conditions produced no effect on the mRNA level of the studied genes coding for NAPs (Figure 5B). We have found that these conditions reduced the mRNA yield of *nahAa, nahU, nahH, catA*(2), and *catA*(8), leaving unchanged *rpoD* mRNA (Figure 3). These results evidence for the existence of other agents whose role in the regulation of naphthalene catabolic gene expression remains unknown.

## Discussion

The regulation of expression of genes involved in the naphthalene-salicylate-catechol-Krebs cycle pathway is studied either in terms of the activation in the presence of inductors (in more detail) or in terms of catabolite repression (in part). Here, the activity of key enzymes of the naphthalene catabolic pathway was studied along with the mRNA level of genes encoding these enzymes in *P. putida* strain BS3701 cultivated in low nitrogen/low iron conditions. In the natural environment, microorganisms have to adapt to nutrient deficiency. In *Pseudomonas*, NtrC positively regulates the transcription of σ54-dependent target genes under low nitrogen conditions, including the expression of *glnA* (Hervás et al., 2009) and *rhlA* (Wenner et al., 2014; Pita et al., 2018), nitrate assimilation operon (Romeo et al., 2012), and nitrogenase genes (Zhan et al., 2019). We observed that mRNAs of *nahU* (salicylate hydroxylase) and *catA* (catechol 1,2-dioxygenase) were down-regulated at decreasing NH_4_Cl concentration from 5 to 1 mM, the *rpoD* mRNA level remained unchanged, but the *ihfA* mRNA level altered. Although IHF has been reported to stimulate the RNAP interaction with specific transcriptional regulators (Hoover et al., 1990), our experiments showed a decreased mRNA level. We assume that *nahU* and *catA* mRNA levels undergo posttranscriptional regulation through the changing mRNA degradation rate. The mRNAs of *nahAa* (naphthalene 1,2-dioxygenase) and *nahH* (catechol 2,3-dioxygenase) remained unchanged, while their enzyme activity changed. Naphthalene 1,2-dioxygenase and catechol 2,3-dioxygenase possibly undergo the posttranscriptional regulation by inhibition/stimulation of translation. The activity of catechol 2,3-dioxygenase decreased. We are the first to report this observation. In contrast, low nitrogen conditions resulted in a higher enzyme activity of naphthalene 1,2-dioxygenase. This effect has not been mentioned in the literature either. Only one fact was found in support of the interconnection between naphthalene 1,2-dioxygenase and nitrogen metabolism. NDO can use indole (a heterocyclic nitrogen-containing compound) as a substrate, having oxidized it to 2,3 dihydroxyindole and further to the salicylate (Ma et al., 2018. It cannot be ruled out that before switching to glutamate catabolism occurring in nitrogen deficiency conditions, *P. putida* BS3701 uses indole.

Fur (Ferric uptake regulator) acts as a repressor under iron-rich conditions (Ochsner et al., 1995), but with deficient iron, in *Pseudomonas*, Fur-dependent ncRNA of PrrF, a RyhB homolog, reduces mRNA stability (Massé et al., 2003; Wilderman et al., 2004). Our study has shown a decrease in the mRNA level of *nahAa*, *nahU*, *nahH*, *catA*(2), and *catA*(8), though that of *rpoD* and NAPs remained unchanged. We assume that the mRNA level undergoes post-transcriptional regulation by changing the mRNA degradation rate. Low iron conditions can produce a critical effect on the functioning of dioxygenases with iron-sulfur clusters (naphthalene 1,2-dioxygenase, catechol 2,3-dioxygenase, and catechol 1,2-dioxygenase). As shown previously (Dinkla et al., 2001), these conditions result in a lower activity of catechol 2,3-dioxygenase. Iron-storage proteins are known to undergo post-transcriptional regulation through an ncRNA-induced increase in the mRNA degradation rate (Massé et al., 2003). The expression of salicylate hydroxylase free of iron-sulfur clusters also varied in response to the decreasing concentration of available iron, which can be a result of interconnection between salicylate and salicylate-derived siderophores [e.g., *Pseudomonas* pyochelin (Crosa and Walsh, 2002) and promysalin (Li W. et al., 2011)]. Annotation using RAST and Prokka revealed that BS3701 contains homologs of genes involved in the synthesis and transport of pyochelin (*fptA, pchR, pchB*; unpublished data).

In *Pseudomonas*, naphthalene catabolism has been best studied in terms of the effects of specific transcription activators. We propose that under low nitrogen and low iron conditions the naphthalene catabolic genes are subject not only to transcriptional but also post-transcriptional regulation. These results form a basis for studying the mechanisms of additional regulatory control of genes involved in aromatic hydrocarbon catabolism.

## Data Availability Statement

The datasets presented in this study can be found in online repositories. The names of the repository/repositories and accession number(s) can be found here: https://www.ncbi.nlm.nih.gov/genbank/, MN442422.1; https://www.ncbi.nlm.nih. gov/genbank/, MN413629.1; https://www.ncbi.nlm.nih.gov/gen bank/, MN442423.1; https://www.ncbi.nlm.nih.gov/genbank/, MN44P2424.1; https://www.ncbi.nlm.nih.gov/genbank/, MN44 2425.1; https://www.ncbi.nlm.nih.gov/genbank/, MN442426.1.

## Author Contributions

IP-F, KP, AV, and MZ contributed to the experiment design. IP-F, KP, AV, AF, and RS conducted all experimental work and analyses. IP wrote the manuscript with critical review and inputs from KP, AV, and MZ.

## Conflict of Interest

The authors declare that the research was conducted in the absence of any commercial or financial relationships that could be construed as a potential conflict of interest.

## Footnotes

1 https://www.ncbi.nlm.nih.gov/tools/primer-blast/
